# Benralizumab Depletes IL‐5Rα‐Bearing Cells in Skin Lesions of Patients With Atopic Dermatitis

**DOI:** 10.1002/clt2.70090

**Published:** 2025-08-08

**Authors:** Christiane E. Whetstone, Ruth P. Cusack, Emma L. Price, Karen J. Howie, Caitlin Stevens, Dhuha Al‐Sajee, Suzanne Beaudin, Jennifer Wattie, Nadia Alsaji, Abbey Schlatman, Vanessa Luk, Paul M. O'Byrne, Mark D. Inman, Roma Sehmi, Hermenio Lima, Gail M. Gauvreau

**Affiliations:** ^1^ Department of Medicine Division of Respirology McMaster University Hamilton Ontario Canada; ^2^ Firestone Institute for Respiratory Health McMaster University Hamilton Ontario Canada; ^3^ Department of Medicine, Division of Allergy and Immunology McMaster University Hamilton Ontario Canada

**Keywords:** atopic dermatitis, basophils, benralizumab, CD125, eosinophils, mast cells

## Abstract

**Background:**

Atopic dermatitis (AD) is a chronic inflammatory skin disease characterized by tissue eosinophilia and itch. We evaluated the effect of eosinophil depletion with benralizumab on markers of inflammation in the skin of patients with AD.

**Methods:**

After a 4‐week washout from oral anti‐inflammatory medications 20 patients with moderate to severe AD completed a randomized, double‐blind, placebo‐controlled parallel‐group study comparing 3 doses q4wk 30 mg subcutaneous benralizumab to placebo. Lesional and unaffected skin biopsies were collected before and after 28 and 65 days of treatment, respectively, for quantification of eosinophils and IL‐5Rα‐bearing cells in papillary dermis. Histological measurements of epidermal thickness, spongiosis, neutrophilic and lymphocytic infiltration, as well as clinical scores EASI, SCORAD, IGA, DLQI, and POEM were conducted throughout the study. Outcomes were compared between placebo and benralizumab treatment groups using a Mann‐Whitney *U*‐test.

**Results:**

Benralizumab, compared to placebo, reduced IL‐5Rα+ cells and MBP+ EG2+ eosinophils in lesions and unaffected skin (*p* < 0.05). In skin lesions, benralizumab reduced MBP+ eosinophils and basophils but had no effect on eosinophil progenitor (EoP; CD34+ IL‐5Rα+) or mast cell numbers. There was no change in other skin histological measurements or IGA scoring of the observational lesion, nor improvement in clinical scores.

**Conclusion:**

Benralizumab treatment significantly inhibited accumulation of MBP+ eosinophils and basophils in lesional skin of patients with moderate to severe AD. However, a lack of improvement in histological and clinical outcomes suggests that other inflammatory pathways are central to the pathobiology of severe atopic dermatitis.

**Trial Registration:**

(ClincialTrials.gov number, NCT03563066)

## 1 Introduction

Atopic dermatitis (AD) affects up to 20% of children and 10% of adults worldwide with 20% of those suffering from severe AD symptoms, characterized by intense itching and widespread inflammation leading to a notably reduced quality of life [1, 2, 3]. While certain key factors such as a compromised skin barrier, susceptibility to environmental triggers and immune dysregulation are recognized as important contributors to disease pathology, the precise interaction(s) of elements are not fully understood. AD is characterized by eosinophilic inflammation and is primarily driven by a type 2 immune response involving innate immune cells and the cytokines interleukin (IL)‐4, IL‐5, IL‐13, and IL‐31.

Benralizumab is an afucosylated monoclonal antibody directed against the alpha chain of the IL‐5 receptor (IL‐5Rα), inducing eosinophil depletion through antibody‐dependent cell‐mediated cytotoxicity (ADCC). Benralizumab is approved for the treatment of severe eosinophilic asthma, another type 2 inflammatory disease [4, 5], and is also found to be effective in other eosinophil prominent diseases such as hypereosinophilic syndrome [6], chronic sinusitis with nasal polyps [7], drug rash with eosinophilia and systemic symptoms (DRESS) [8], eosinophilic granulomatosis with polyangiitis [9], and eosinophilic pustular folliculitis [10].

Eosinophils are recruited to inflamed tissues by the potent chemoattractants eotaxin‐1, eotaxin‐3 and RANTES [11, 12]. Once activated, eosinophils release toxic granule proteins as well as cytokines and chemokines, including IL‐6, IL‐12, TGF‐β1 and IL‐13 [13, 14, 15]. Altogether, these mediators contribute to tissue fibrosis [16], edema with blister formation [17, 18], and pruritus [19]. Type 2 cytokine stimulation of eosinophils can lead to the release of IL‐12, which in turn promotes a switch from type 2 inflammation typical of acute lesions to a type 1 immune response characteristic of chronic AD lesions [20, 21].

The presence of eosinophils in the cutaneous inflammatory infiltrates of AD skin lesions has long been established, however the role of these cells remains unclear. To understand the role of eosinophils in the pathobiology of chronic AD lesions, we conducted a 9 weeks, double‐blind, placebo‐controlled phase 2b study of benralizumab treatment in patients with moderate‐to‐severe AD. The effects of benralizumab on IL‐5Rα‐bearing cells in blood and on skin inflammation following intradermal challenge in these patients has been reported previously [22].

## 2 Methods

### 2.1 Patients

Eligible consenting patients were male (*n* = 9) and female (*n* = 11), 18–65 years of age, with moderate‐to‐severe AD as determined by EASI score > 7, with disease not adequately controlled by topical corticosteroids, but able to withhold oral anti‐inflammatory medications such as systemic corticosteroids, cyclosporine, mycophenolate‐mofetil, azathioprine or antihistamines for the duration of the study. Co‐morbid asthma was permitted and identified in a subset of patients using medical history (Table 1).

**TABLE 1:** Baseline demographics and clinical characteristics of patients that completed up to Day 65 assessment.

	Total (*n* = 20)	Placebo (*n* = 11)	Benralizumab (*n* = 9)
Race			
White	14	9	5
Black	2	0	2
Asian	3	1	2
South Asian	1	1	0
Age	39.5 (± 3)	38.7 (± 4.2)	40.9 (± 4.6)
Sex			
Male	9	4	5
Female	11	7	4
Height (cm)	171.3 (± 2.1)	168 (± 2.9)	175.2 (± 2.8)
Weight (kg)	125.8 (± 11.0)	127.9 (± 15.3)	123.3 (± 16.9)
Age of AD diagnosis	11.6 (± 3.8)	11.6 (± 5.3)	11.6 (± 5.7)
Disease severity			
EASI score	22.7 (± 3.1)	22 (± 4.1)	23.7 (± 5)
Moderate AD	11	6	5
Severe AD	9	5	4
Asthma diagnosis	14	7	7
Blood eosinophils (10^6^/mL)	0.42 (± 0.08)	0.43 (± 0.11)	0.41 (± 0.12)

*Note:* Data are shown as mean (± SEM).

### 2.2 Study Design

**FIGURE 1:**
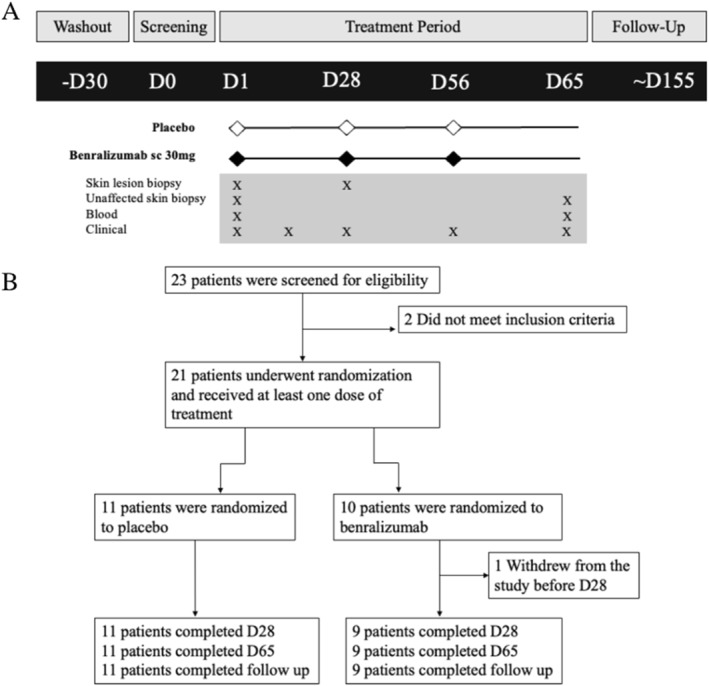
(A) Study Design. Patients were randomized 1:1 to receive benralizumab (30 mg sc monthly) or placebo at Days 1, 28 and 56. Lesion skin biopsies were collected on Days 1 and 28. Blood and unaffected skin biopsies were collected on Days 1 and 65. An observational skin lesion was evaluated on Days 1, 14, 28, 56, and 65. (B) CONSORT diagram summarizing the flow of patients through, randomization, treatment, and follow‐up.

This randomized, double‐blind, parallel group, placebo‐controlled study evaluated the effect of 3 doses of 30 mg benralizumab administered subcutaneously every 4 weeks on measures of inflammation and clinical scores of AD (Figure 1A). After a 4‐week washout from oral anti‐inflammatory medications patients entered a screening period. Those meeting eligibility completed a baseline visit at Day 1 for sampling of pre‐dosing blood as well as punch biopsies from lesional and unaffected skin obtained from a standardized location on the mid‐low section of the back. This site was chosen to accommodate the multiple biopsies required and to ensure the comfort of patients. Blood and unaffected skin biopsies located contralateral to pre‐dosing samples were collected again at Day 65 end of treatment visit, and skin lesions contralateral to pre‐dosing samples were collected earlier, at Day 28, to ensure an adequate lesion was available for histological and cellular analysis. In the two cases where contralateral lesions were not present on the mid‐low back, punch biopsies were collected from the hands. Investigator assessments of disease severity and patient‐reported outcomes were conducted at Day 1 pre‐dosing, and Days 14, 28, 56, and 65. An observational skin lesion was identified for evaluation by IGA on Day 1 and reassessed throughout the study at Days 14, 28, 56, and 65. All clinical and laboratory procedures were conducted at McMaster University, Hamilton, Ontario, Canada.

### 2.3 Study Endpoints

The main objective was to evaluate the effect of benralizumab on the number of IL‐5Rα‐bearing cells, namely eosinophils, eosinophil progenitor cells, basophils, and mast cells in skin lesions at Day 28 post‐treatment, compared to placebo. Exploratory endpoints evaluated skin lesions and unaffected skin for the effect of benralizumab on IL‐5Rα‐bearing cells, and histological measurements of epidermal thickness, spongiosis, neutrophilic and lymphocytic infiltration in lesions and unaffected skin, compared to placebo. We also evaluated the effect of benralizumab on clinical endpoints of disease severity by comparing changes in Eczema Area and Severity Index (EASI), SCORing Atopic Dermatitis (SCORAD), Investigator Global Assessment (IGA), Dermatology Life Quality Index (DLQI), and Patient Oriented Eczema Measure (POEM) scores.

### 2.4 Immunofluorescence Staining and Microscopy

Skin biopsies were formalin‐fixed and embedded into paraffin blocks. Tissue sections were stained by hematoxylin and eosin, and by indirect immunofluorescence microscopy with antibodies binding to activated eosinophil cationic protein (EG2 clone), major basic protein (MBP), IL‐5Rα (CD125), hematopoietic progenitor cells (HPC; defined as CD34+ Von Willebrand factor‐cells), eosinophil progenitor cells (EoP; defined as CD34+ CD125+ Von Willebrand factor‐cells), basophil identified by positive basogranulin (2D7 antibody) immunostaining and mast cells by positive tryptase immunostaining.

### 2.5 Flow Cytometry

Blood was collected for cell enumeration by flow cytometry at Day 1 and Day 65. Eosinophil counts were performed by CBC report, and peripheral blood mononuclear cells isolated by density gradient separation from whole blood were subject to immunofluorescent staining to enumerate HPC, EoP, and type 2 innate lymphoid cells (ILC2s) as previously described by Whetstone et al. 2024 [22]. Peripheral blood mononuclear cells were subject to immunofluorescent staining for CD4+ T cells (CD45+ Lin+ CD4+), and CD4+ CD125+ T cells (CD45+ Lin+ CD4+ CD125+), as detailed in the Supporting Information S1.

### 2.6 Histological Measurements

Epidermal thickness was measured by calculating the region of interest (total area of epidermis) divided by the length using the Nikon imaging software. Evaluation of epidermal spongiotic vacuoles and spongiosis was conducted on a severity scale of 0–3 (0 = none present, 1 = haloing present, 2 = haloing present with vacuoles formed, 3 = numerous vacuoles with intravacuolar lymphocytic or neutrophilic infiltrate). Evaluation perivascular neutrophilic and lymphocyte infiltrate was conducted using a severity scale from 0 to 3 (0 = none present, 1 = few/no inflammatory cell present, 2 = grouping of immune cells not associated with a hair follicle, 3 = intense/diffuse presentation of infiltration).

### 2.7 Statistical Analyses

Statistical analysis was performed on all patients that completed up to Day 65 per protocol. Mann‐Whitney *U*‐test was used to compare the pre to post treatment delta between benralizumab and placebo groups for cellular outcomes measured by microscopy, and flow cytometry, and clinical scores (EASI, SCORAD, IGA, DLQI, POEM). Patients were also divided based on asthma diagnosis and baseline blood eosinophil levels (cells/μL), and these groups were compared using Mann‐Whitney *U*‐test. Correlations between groups were performed using Spearman correlation.

For detailed clinical and laboratory methods, see the Supporting Information S1.

## 3 Results

### 3.1 Study Population

Of 23 patients with AD who were screened, 21 met eligibility criteria and were randomized (Figure 1B). One patient randomized to benralizumab withdrew from the study due to an increase in disease severity. There were no significant differences in baseline characteristics between the benralizumab and placebo groups (Table 1). 11 patients had moderate AD and 9 had severe AD as determined by Eczema Area and Severity Index (EASI). The majority of the study patients had a diagnosis of asthma. The absolute number of blood eosinophils was significantly higher in patients with comorbid asthma compared to those without asthma (Supporting Information S1: Figure 1A), but no difference in EASI score. However there was a trend for EASI score to be higher in patients with high baseline blood eosinophils (> 0.3 cells/μL) compared to AD patients with low baseline blood eosinophils (≤ 0.3 cells/μL) (*p* = 0.065) (Supporting Information S1: Figure 1B) and this was driven by a significant positive association between EASI and blood eosinophils in the asthma group (*p* = 0.04) (Supporting Information S1: Figure 1C). There were no drug‐related adverse events or serious adverse events during the study.

### 3.2 Effect of Benralizumab Treatment on Eosinophils and Progenitor Cells

**FIGURE 2:**
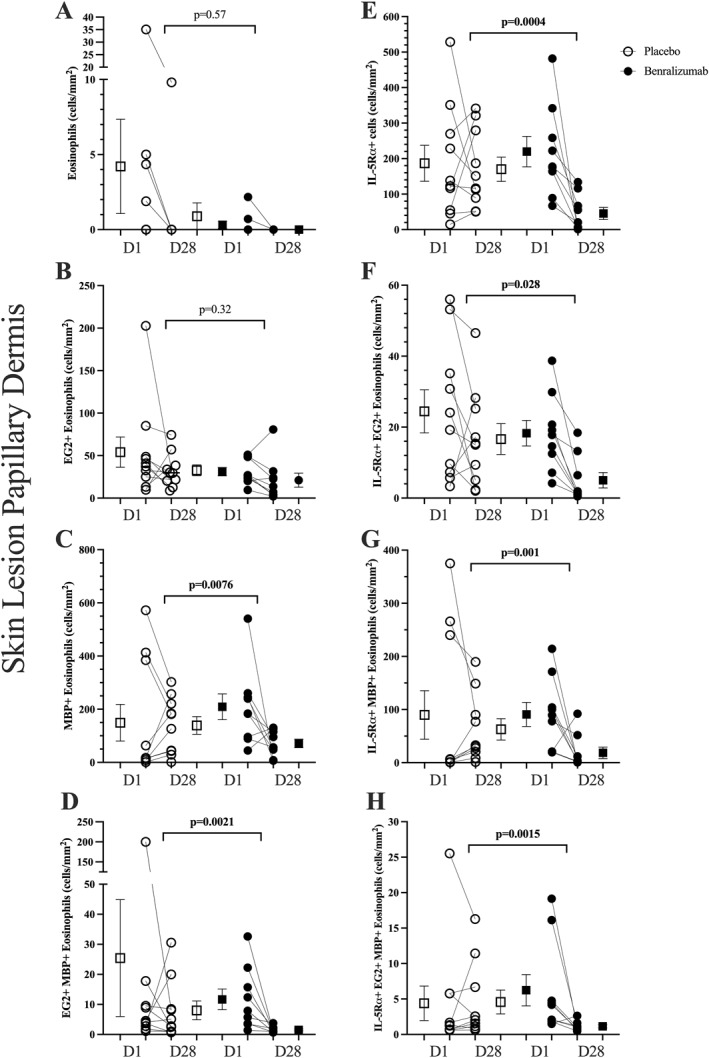
The effect of benralizumab compared to placebo on the number of eosinophils in papillary dermis of skin lesions measured by (A) H&E, (B) EG2+, (C) MBP+, and (D) EG2+ MBP+; (E) cells expressing IL‐5Rα and eosinophils co‐expressing IL‐5Rα in papillary dermis of lesion skin measured by (F) CD125+ EG2+, (G) CD125 + MBP+, and (H) CD125+ EG2+ MBP+. Data are shown as individual and mean (SEM), the delta change from pre to post treatment measurements were compared between benralizumab and placebo groups by Mann‐Whitey *U* test.

After 65 days of treatment, there was a complete depletion of blood eosinophils in the benralizumab group, compared to no change in the placebo group (*p* < 0.0001) as previously reported [22]. In skin lesions at Day 28 benralizumab significantly reduced the number of MBP+ (*p* = 0.0076), IL‐5Rα+ cells (*p* = 0.0004), eosinophils double positive for IL‐5Rα+ EG2+ (*p* = 0.028) and IL‐5Rα+ MBP+ cells (*p* = 0.001), and eosinophils triple positive for IL‐5Rα+ EG2+ MBP+ (*p* = 0.0015) compared to placebo (Figure 2). In contrast there was no change in tissue eosinophils stained using hematoxylin and eosin or immunofluorescence staining of EG2 (*p* = 0.57 and *p* = 0.32, respectively). Representative staining is shown in Supporting Information S1: Figure 2. In skin lesions at Day 28 benralizumab treatment had no effect on the number of HPC (*p* = 0.5) and EoP (*p* = 0.72) compared to placebo (Figure 2).

**FIGURE 3:**
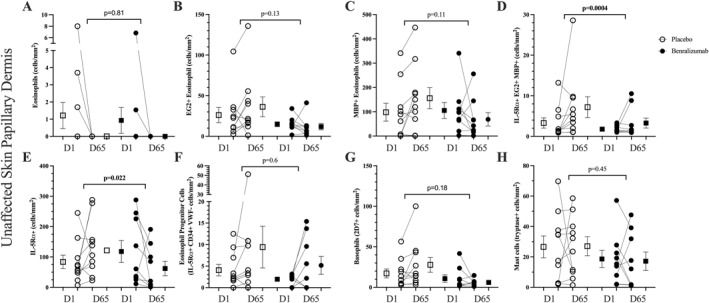
The effect of benralizumab compared to placebo on eosinophils in papillary dermis of unaffected skin measured by (A) H&E, (B) EG2+, (C) MBP+ and (D) EG2+ MBP+; (D) eosinophils co‐expressing CD125+ EG2+ MBP+; (E) cells expressing CD125+; eosinophil progenitor cells defined as CD34+ CD125+ Von Willebrand Factor−; (G) basophils (2D7+ cells); (H) mast cells (tryptase+). Data are shown as individual and mean (SEM) and analyzed by Mann‐Whitey *U* test.

In the unaffected skin at Day 65, benralizumab significantly reduced the number of IL‐5Rα+ EG2+ MBP+ eosinophils (*p* = 0.0004) and total IL‐5Rα+ cells compared to placebo (*p* = 0.022) but there was no effect of benralizumab on eosinophil numbers detected by chemical staining with hematoxylin and eosin (*p* = 0.81) or immunostaining with EG2 (*p* = 0.13) or MBP (*p* = 0.11), or on EoP cell numbers (*p* = 0.6) (Figure 3).

### 3.3 Effect of Benralizumab Treatment on Basophils and Mast Cells

**FIGURE 4:**
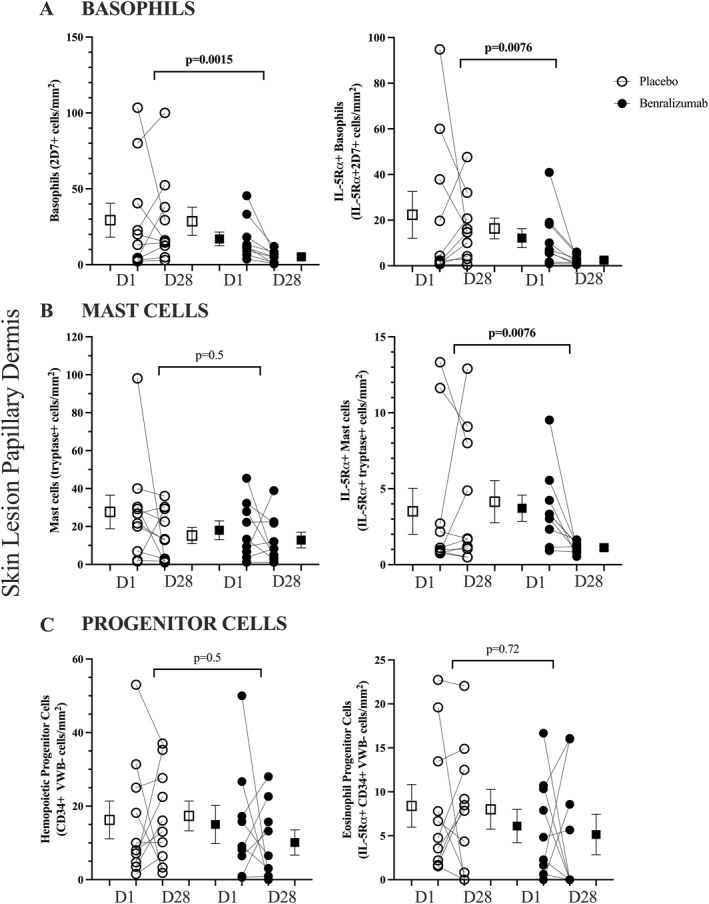
The effect of benralizumab compared to placebo on the number of (A) basophils (2D7+ cells) and (B) mast cells (tryptase+) and (C) progenitor cells (CD34+ Von Willebrand Factor‐) with and without co‐expression of IL‐5Rα in papillary dermis of skin lesions. Data are shown as individual and mean (SEM), the delta change from pre to post treatment measurements were compared between benralizumab and placebo groups by Mann‐Whitey *U* test.

In skin lesions at Day 28, benralizumab significantly reduced the number basophils (2D7+ cells; *p* = 0.0015), IL‐5Rα+ basophils (CD125+ 2D7+ cells; *p* = 0.0076), IL‐5Rα+ mast cells (CD125+ tryptase+ cells; *p* = 0.0076) with no change in the number of mast cells (tryptase+ cells; *p* = 0.5) compared to placebo (Figure 4). Representative staining is shown in Supporting Information S1: Figure 3. In unaffected skin there was no effect of benralizumab on the number of basophils or mast cells (Figure 3).

**FIGURE 5:**
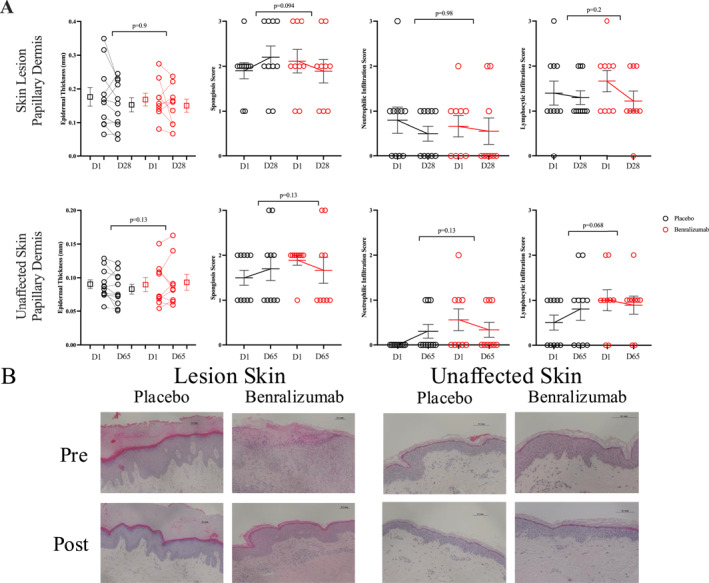
The effect of benralizumab compared to placebo on (A) epidermal thickness, spongiosis, neutrophilic and lymphocytic infiltration of lesion and unaffected skin. (B) Representative images of lesion and unaffected skin biopsies before and after treatment in the placebo and benralizumab treatment arms.

Benralizumab did not reduce the frequency of CD4+ T cells (*p* = 0.76) or CD4+ T cells expressing CD125 (*p* = 0.84) in blood at Day 65 (Supporting Information S1: Figure 4); Flow cytometric logical gating for T cells is shown in Supporting Information S1: Figure 5.

### 3.4 Effect of Benralizumab on Histological Measurements

At Day 28, patients in the benralizumab group demonstrated trends toward a lower spongiosis score in skin lesions (*p* = 0.094), compared to placebo; however, there was no change in measurements of epidermal thickness, neutrophilic or lymphocytic infiltration (Figure 5A). At Day 65, the benralizumab group trended toward lower lymphocytic infiltration in unaffected skin (*p* = 0.065), compared to placebo, with a numerical but not statistically significant decrease in spongiosis and neutrophilic infiltration (Figure 5A). Representative images are shown in Figure 5B.

### 3.5 Effect of Benralizumab on Clinical Outcomes and Patient Questionnaires

Clinical assessments of AD disease severity showed no significant differences between benralizumab and placebo treatment for SCORAD, IGA, DLQI and POEM assessments (*p* = 0.94, *p* = 0.11, *p* = 0.59, *p* = 0.84, respectively) (Supporting Information S1: Figure 6), and observations of the skin lesion assessed longitudinally by IGA score did not improve either (*p* = 0.56) (data not shown). Even though we observed a trend for higher EASI scores in patients with high baseline blood eosinophils (> 0.3 cells/μL) compared to AD patients with low baseline blood eosinophils (≤ 0.3 cells/μL) (*p* = 0.065) (Supporting Information S1: Figure 1B), when we examined only patients with high blood eosinophil levels (> 0.3 cells/uL), there was no indication that clinical assessments by EASI, SCORAD, IGA, DLQI and POEM were improved by benralizumab (*p* = 0.73, *p* = 0.11, *p* = 0.1, *p* = 0.72, *p* = 0.67, respectively) (Supporting Information S1: Figure 7).

## 4 Discussion

Benralizumab, an anti‐IL‐5Rα monoclonal antibody, significantly depleted blood and lesional skin MBP+ eosinophils in adults with moderate‐to‐severe atopic dermatitis. Benralizumab also significantly reduced basophil levels but showed no reduction in mast cells, no improvements in skin histological scores, clinical outcomes, or patient reported clinical outcomes. These findings are consistent with a larger phase 2 clinical trial showing no change in the IGA and EASI clinical scores or itch improvement after 16 weeks of benralizumab treatment in moderate‐to‐severe AD. [23].

Interestingly, tissue eosinophils identified by hematoxylin and eosin staining and EG2 immunostaining were not reduced in lesional skin, while tissue eosinophils identified by MBP and all eosinophil sub‐types expressing CD125 were significantly attenuated. Although the presence of eosinophils in the inflammatory infiltrate of AD has long been established, the precise role of eosinophils, and in particular different eosinophil sub‐types, in this disease remains unknown and requires further investigation.

Blood eosinophil counts have been roughly correlated with AD disease severity, though many patients with severe disease have normal peripheral blood eosinophil counts [24]. In fact, patients with normal eosinophil counts mainly present with atopic dermatitis alone, whereas those with severe atopic dermatitis alongside respiratory allergies and allergen sensitization frequently display heightened levels of peripheral blood eosinophils [25]. In our population of AD patients, we found patients with elevated eosinophil levels (> 0.3 cells/μL) tended to have increased AD disease severity as measured by higher EASI score, and patients with comorbid asthma had significantly higher eosinophil levels than the non‐asthmatic patients. Additionally, in the AD patients with asthma, baseline eosinophil levels were significantly correlated with AD disease severity (*r* = 0.56, *p* = 0.04).

In patients with asthma, it is now well established that anti‐IL‐5 therapy is more effective in those with elevated blood (> 150 cells/μL) and sputum (> 3%) eosinophil levels [26, 27]. Our study did not recruit AD patients based on blood eosinophil levels. To understand if AD patients with high eosinophil levels respond to benralizumab treatment, we performed a subgroup analysis in patients with blood eosinophil levels > 0.3 cells/μL. Although underpowered, we found EASI, SCORAD, IGA, DLQI and POEM scores in these selected patients were similar to the analyses of the entire group, suggesting that high blood eosinophil levels in atopic dermatitis are not predictive of an improved response to benralizumab, nor do they likely contribute to a clinical response to benralizumab.

Circulating eosinophils have been observed in AD patients since the first descriptions of the disease [28]. In chronic lesions, however, intact eosinophils are sparse, while elevated levels of eosinophil granule proteins in the tissue and blood suggest that infiltrating eosinophils are activated and quickly degranulate [29]. This might explain the low number of eosinophils in the lesion biopsies identifiable by hematoxylin and eosin staining compared to immunostaining with MBP and EG2. Comparing lesions and unaffected skin with each stain, we were surprised to find similar levels of eosinophils. High eosinophil levels have also been reported in the unaffected skin of patients with chronic spontaneous urticaria, possibly signifying that the skin is primed for further inflammatory cell infiltration [30, 31]. As inflammation progresses from acute to chronic, it is possible that the degranulation of eosinophils presents a technical challenge when relying on eosinophil granule expression for quantification, which may contribute to our inability to detect changes in H&E‐stained eosinophils in lesion biopsies after benralizumab treatment.

Given the evidence suggesting that eosinophil granules rather than intact eosinophils contribute to atopic dermatitis pathogenesis [32, 33], we categorized eosinophils by their expression of eosinophil granular proteins, including major basic protein (MBP) and activated eosinophil cationic protein (ECP), aggregated in the papillary dermis of the biopsies. The EG2 antibody recognizes the activated form of ECP, and therefore, EG2+ eosinophils represent cells that have been activated and primed for the secretory exocytosis process [34, 35], whereas anti‐MBP stains all eosinophils irrespective of their degree of activation [36]. We found that MBP+ eosinophils in lesions were significantly reduced with benralizumab treatment compared to placebo, whereas EG2+ levels were unchanged. Interestingly, this is opposite to what was found in allergen‐challenged skin, where EG2+ eosinophils were significantly reduced with benralizumab treatment, but MBP+ eosinophils were not [22]. We have shown that the majority of MBP+ eosinophils express CD125, whereas a smaller proportion of EG2+ eosinophils express CD125, providing a rationale for why MBP+ eosinophils, but not EG2+ eosinophils were reduced by benralizumab. In the skin of patients with chronic urticaria, MBP staining is reported to be distributed diffusely throughout the tissue, whereas staining of ECP by EG1 and EG2 antibodies were found close to individual cells [31]. These differential degranulation patterns may also be occurring in lesional skin of AD and contributing to our ability to detect intracellular MBP and ECP. The differential responses of these eosinophil subsets to benralizumab may also be a result of their inflammatory microenvironment, with peripheral blood and acute lesions dominated by type 2 inflammation, and chronic lesions dominated by type 1 and type 17/22 inflammatory responses [37].

When EG2+ and MBP+ eosinophils were assessed for co‐expression with IL‐5Rα (CD125), a significant reduction was observed in both EG2+ CD125+ cells and MBP+ CD125+ cells, as would be expected after benralizumab treatment. Although the interpretation of these results suggests that CD125+ eosinophils do not contribute to the pathobiology of AD, it is important to note that not all eosinophils in the tissue express CD125 as reported in allergen‐challenged skin of AD patients [22] and airways of asthmatic patients [38, 39, 40]. Studies in asthmatics indicate that the expression of CD125 can differ between eosinophils found in the blood from those found in the airways (sputum), suggesting that the variation in CD125 expression might be related to eosinophil activation, migration, and potentially serve as a mechanism to prevent excessive eosinophil activation by IL‐5 in the airways [41]. Although this has not been directly studied in AD, observations from the airways of asthmatic patients may help explain why only a portion of eosinophils found in the dermis express CD125. Furthermore, it is unknown whether a subpopulation of tissue eosinophils that are IL‐5‐independent contributes to the pathobiology of allergic diseases in humans [42, 43]. In asthmatic patients treated with mepolizumab, the expression of receptors for eosinophil growth factors IL‐3, IL‐5, and GM‐CSF on airway eosinophils is not altered by mepolizumab, indicating a population of eosinophils that can be readily activated without IL‐5 [42]. Further exploration is required in the context of skin inflammation.

In conjunction with eosinophils, other important immune cells such as basophils, activated B cells, ILC2s, and mast cells—highlighted in this study—are identified as expressing CD125 [44, 45, 46]. As previously reported by our group, in blood at Day 65, benralizumab significantly reduced the frequency of ILC2s, ILC2 cells positive for CD125 and EoP cells in blood compared to placebo, but had no effect on the number of HPCs [22]. There was a significant reduction in basophils identified as 2D7+ cells and basophils expressing IL‐5Rα identified as 2D7+ CD125+ cells in lesion skin. There was no difference in the number of mast cells identified by tryptase alone (*p* = 0.5), whereas the subset of mast cells expressing IL‐5Rα was significantly reduced in lesion skin by benralizumab. Our data show that in the skin of AD patients, roughly 70% of basophils express IL‐5Rα, whereas approximately 35% of mast cells express IL‐5Rα. Despite the near depletion of basophils and eosinophils by benralizumab, a relatively large population of mast cells remaining in both acute and chronic lesions degranulate and release cytokines, 5‐HT, histamine and nerve growth factor, which may continue to drive intense pruritus and contribute to disease symptoms [47, 48]. Although histamine‐driven pruritus doesn't seem to substantially contribute to chronic itch in AD [49], mast cell‐directed therapies have been particularly interesting, including lirentelimab (anti‐Siglec‐8) and kit‐inhibitors in patients with chronic urticaria or allergic conjunctivitis [50, 51]; reducing this CD125+ sub‐population of mast cells provides an alternate mechanism of action compared to these other therapies under clinical development to target mast cells.

Keratinocytes and CD4+ T cells can release enough pruritogenic cytokines (IL‐4, TSLP, IL‐31 and substance P) to make up for the reduction in eosinophils and basophils [52, 53, 54, 55]. The number of CD4+ T cells and CD4+ T cells expressing CD125 in circulation were unaffected by benralizumab treatment. CD4+ T cells may differentiate into a variety of T cell subsets, including Th1, Th2, Th17 and Treg, all of which are key components in orchestrating the adaptive immune response. Given the wide range of pro‐inflammatory pathways initiated by T cells, it is possible these cells continue to drive AD symptoms and severity during benralizumab treatment.

Biologics and Janus kinase inhibitors (JAKi) targeting upstream components of the inflammatory cascade are increasing our understanding of the pathobiology of AD. Dupilumab (an anti‐IL‐4Rα monoclonal antibody) has been proven to be effective in multiple allergic diseases, including both allergic asthma and AD [56, 57]. Dupilumab binds to the IL‐4Rα, which is shared by both IL‐4 and IL‐13, inhibiting the T2 inflammatory cascade with a broader range than targeting IL‐5. Additionally, CD4+ T cells are known to directly interact with keratinocytes, inhibiting differentiation markers and favoring itch notably through the release of cytokines IL‐4/IL‐13/IL‐31, leading to barrier disruption, which in turn induces secretion of alarmins TSLP, IL‐33 and IL‐25 by keratinocytes [54, 58, 59, 60, 61]. Other biologics targeting IL‐13 including tralokinumab and lebrikizumab have been successful at reducing itch and improving quality of life scores in AD patients [62, 63]. Nemolizumab, which targets the IL‐31 receptors found on nerve cells and eosinophils driving the itch response, has been effective at decreasing itch [64]. In contrast, biologics targeting IL‐17c [65] and IL‐33 [66] have failed in AD clinical trials. The role of JAKi in modulating eosinophil function in AD needs further investigation.

The positive relationship between eosinophils and the EASI score has provided indirect evidence for a role of eosinophils in the propagation of AD. However, we and others have now confirmed that depleting eosinophils in blood and significantly reducing eosinophils in tissue has no effect on clinical scores in patients with moderate‐to‐severe AD. Benralizumab demonstrates strong efficacy in treating severe eosinophilic asthma and eosinophilic esophagitis (EoE), conditions in which disease pathology is largely driven by an overabundance of eosinophils and their activation via the IL‐5 pathway. In contrast, while eosinophils contribute to the inflammatory milieu of AD, they are neither as predominant nor as central to its pathogenesis. Instead, AD reflects a complex immunologic landscape involving T cells, mast cells, and eosinophils. Consequently, although benralizumab may reduce eosinophil levels in AD, its overall therapeutic benefit is likely limited, given the multifaceted nature of the disease and the involvement of multiple overlapping immune pathways. Perhaps there is a subset of accessory eosinophils helping to activate and polarize T cell proliferation into Th2 cells and inducing their release of pruritogenic cytokines, as well as causing direct injury to the epidermis and subsequent release of pruritogenic cytokines from keratinocytes [67]. However, the exact interaction between eosinophil subsets and the epidermis requires future investigation.

## 5 Conclusion

Benralizumab significantly attenuated eosinophil levels in lesional skin of patients with moderate‐severe atopic dermatitis but these changes were not accompanied by improved skin histological or clinical scores.

## Conflicts of Interest

P.M.O. reports research funding from AstraZeneca, Merk and Biohaven paid to the institution, consulting fees from AstraZeneca, GSK, Sage, Teva, Affibody, and lecture fees from AstraZeneca, Chiesi, GSK. R.S. reports research funding from AstraZeneca paid to the institution. G.M.G. reports research funding from AstraZeneca, Genentech, Third Harmonics Bio, and Biohaven paid to the institution, and lecture fees from AstraZeneca.

## Supporting information

Supporting Information S1

## Data Availability

The data that support the findings of this study are available on request from the corresponding author. The data are not publicly available due to privacy or ethical restrictions.
