# Supplementary material for: Benralizumab Depletes IL‐5Rα‐Bearing Cells in Skin Lesions of Patients With Atopic Dermatitis
Source: Clin Transl Allergy. 2025 Aug 8;15(8):e70090. doi: 10.1002/clt2.70090 (PMC12334360; doi:10.1002/clt2.70090)
Supplement: Supplementary file 1 — Supporting Information S1 [file CLT2-15-e70090-s001.docx]

**Benralizumab depletes IL-5Rα-bearing cells in skin lesions of patient with atopic dermatitis**

**Online Supplemental Data**

**Methods**

*Study design*

This randomized, double-blind, parallel group, placebo-controlled study evaluated the effect of 3 doses of a fixed 30 mg dose of benralizumab administered subcutaneously (SC) every 4 weeks to patients with moderate-to-severe atopic dermatitis, on the severity of atopic dermatitis, and the cellular inflammation of skin lesions in these patients.

***Screening and Baseline***

During a screening period (Days -1 to 0), patients with moderate-to-severe atopic dermatitis who developed late phase cutaneous response following intradermal allergen challenge, were recruited for the study. 11 patients had moderate AD severity and 9 had severe AD as determined by Eczema Area and Severity Index (EASI) with scoring of skin categorized as 0 = clear, 0.1-1.0 = almost clear, 1.1-7.0 = mild, 7.1-21.0 = moderate, 21.1-50.0 = severe, 50.1-72 = very severe.

***Effect of treatment on clinical disease***

Biopsies from skin lesions and samples of peripheral blood were examined for levels of cells that express IL-5Rα subunit including eosinophils, basophils, eosinophil progenitor cells, and innate lymphoid type 2 cells using flow cytometry. In addition, methylcellulose colony forming assay enumerated the IL-5 driven clonogenic potential of progenitor cell populations within the blood.

***Effect of treatment on allergen-induced responses***

An intradermal allergen challenge was conducted 1 week after the last dose of study drug to determine the effect of benralizumab on allergen-induced responses in skin. The size of the resulting skin wheal was measured at 24h post-challenge. Biopsies of the skin wheal were obtained for measurements of eosinophils and basophils per mm^2^ tissue using standard histochemical and immuno-histochemical stains. Additional biopsies of the skin wheal were collected to measure the frequency of eosinophils, basophils, eosinophil progenitor cells, and innate lymphoid type 2 cells by flow cytometry.

***Follow-up***

Patients returned for a safety follow-up visit on study Day 140 (week 20), which is 12 weeks after the last dose/11 weeks after the last study procedure.

*Clinical Procedures*

***Skin Prick Test***

A skin prick test was used to determine the allergen(s) to which each subject is sensitized. Standard allergen extracts included ragweed, trees, grass, dog, cat, horse, dust mites (Dermatophagoides *farinae* and D. pteronyssinus), Alternaria, and Aspergillus. Extracts were applied to the back then pricking the skin to allow exposure, and then evaluating the local reaction in the skin. A positive control (1 mg/mL histamine) and a negative control (diluent) were applied to the skin. If an allergen provoked an allergic reaction, a raised itchy bump (wheal) developed. The size of the wheal (the raised area, not the redness) was measured and recorded with a ruler in millimeters in the horizontal and vertical directions, perpendicular to each other after approximately 15 minutes. The size of the wheal for each antigen was recorded, along with any observed adverse reaction or event and any actions taken. A reaction greater than 2x2 mm was regarded as positive, provided that the positive and negative controls were appropriately positive (histamine) and negative (diluent), respectively.  Based on skin prick and allergen specific IgE blood RAST test, the investigator chose an allergen for skin prick titration. The selected allergen was diluted 2, 4, 8, 16, 32, 64, 128, and 256-fold with normal saline and an additional skin prick test was performed. After 10 minutes, the size of the wheal was measured. The dilution that resulted in a wheal size of 3x3 mm was selected for intradermal allergen challenge. This procedure was completed in accordance with the allergen skin testing using the epicutaneous method SOP at McMaster University (Hamilton, Ontario, Canada).

Allergen extracts manufactured following GMP guidelines, were selected, prepared by staff and administered to the subjects by injection in accordance with procedures approved by Health Canada.

***Skin Sampling***

Punch biopsies were obtained from the site of the intradermal allergen and saline challenges using a sterile 4 mm skin punch by applying and twisting until the blade of the skin punch has pierced the skin. The biopsy was removed using sterile forceps and a scalpel. These samples were processed for histologic examination of inflammatory cells, including eosinophils, mast cells, basophils supernatant cytokine levels. Before a biopsy was taken, the skin was thoroughly cleaned, and local anesthetic (2% lidocaine) is injected to numb the skin. The site of skin biopsies and excision was sutured and covered with a sterile bandage. Patients were instructed how to keep the site clean, and sutures were removed at the next study visit.

***Blood Sampling***

At visit 1 (D-1, screening) and visit 7 (D140) blood was collected for safety labs including complete blood count, coagulation, and chemistry including alanine aminotransferase and aspartate aminotransferase. At visit 1 blood was also collected for virology (hepatitis B and C, and HIV). Incidental findings would be given the appropriate medical follow up. Women had blood drawn for pregnancy testing or FSH to confirm post-menopausal status. At visits 2 (D0) and 6 (D65), 30 mL of blood was drawn from a vein in the arm using aseptic techniques, to measure circulating levels of inflammatory cells including eosinophils, eosinophil progenitor cells (EoP), hematopoietic progenitor cells (HPCs), and innate lymphoid cells (ILC2). Those performing patient and laboratory assessments remained blinded to the results of the blood cell counts.

***Urine Sampling***

At visit 1 (screening) and visit 8 (follow up) urine was collected for safety urinalysis. After enrolment, urine was collected from women at visits 2, 3 and 4 before dosing for urine pregnancy testing.

***Eczema Area and Severity Index***

The EASI is a validated measure used in clinical practice and clinical trials to assess the severity and extent of atopic dermatitis. It is a composite index with scores ranging from 0 to 72. Four atopic dermatitis disease characteristics (erythema, thickness [induration, papulation, edema], scratching [excoriation], and lichenification) were each assessed for severity by the investigator or designee on a scale of “0” (absent) through “3” (severe). In addition, the area of AD involvement was assessed as a percentage by body area of head, trunk, upper limbs, and lower limbs, and converted to a score of 0 to 6. In each body region, the area is expressed as 0, 1 (1% to 9%), 2 (10% to 29%), 3 (30% to 49%), 4 (50% to 69%), 5 (70% to 89%), or 6 (90% to 100%).

*Laboratory Procedures*

***Immunofluorescence and immunohistochemistry (IF and IHC) and* *Imaging***

Tissues were formalin-fixed, embedded into paraffin blocks and H&E stained by the McMaster Core Histology Laboratory Facility. Tissue sections were stained by indirect immunofluorescence for eosinophil cationic protein EG2, major basic protein (MBP), CD125, eosinophil progenitor cells (CD34+ CD125+ Von Willebrand-), basophils (2D7) and mast cells (tryptase). Paraffin-embedded tissues were cut into 5um sections and mounted on frosted positively charged glass slides. Sections were deparaffinized in xylene and rehydrated with subsequent reductions in decreasing concentration of ethanol and subjected to heat-induced antigen retrieval in sodium citrate buffer (pH=6) under pressure for 5 minutes. Permeabilization of cells was performed using 0.2% trition X-100 for 30 minutes. For assessing mature eosinophils in tissues, biopsies were stained for EG2, MBP and CD125 (Supplementary Figure 1). CD125 staining was performed by using 3% normal horse blocking serum in PBS for 40 minutes and incubated with primary antibody goat anti-human CD125 (PA5-47340; Invitrogen, MS, United States; dilution 1:250) overnight at 4°C and detected using a donkey anti-goat Alexa Fluor 594 (A11058; Invitrogen, MS, United States) for 1 hour at room temperature. EG2 staining was performed by using 1.5% normal goat blocking serum and 1.5% normal horse blocking serum in PBS for 30 minutes and incubated with primary antibody mouse anti-human EG2 (Mab593; Diagnostics Development, Uppsala, Sweden; dilution 1:100) overnight at 4°C and detected using a goat anti-mouse Alexa Fluor 488 (A11001; Invitrogen, MS, United States) for 1 hour at room temperature. MBP staining was performed by using 1.5% normal goat blocking serum and 1.5% normal horse blocking serum in PBS for 30 minutes and incubated with primary antibody rabbit anti-human MBP (ab187523; abcam, Cambridge, United Kingdom; dilution1:200) overnight at 4°C and detected using a goat anti-mouse Alexa Fluor 647 (A21244; Invitrogen, MS, United States) for 1 hour at room temperature. Tissues were counter stained with DAPI and cover slipped.

For eosinophil progenitor cells, cells were identified as being CD34+ CD125+ and Von Willebrand negative (Supplementary Figure 1). Staining was performed using 3% normal horse blocking serum in PBS for 30 minutes and incubated with primary antibody goat anti-human CD125 (PA5-47340; Invitrogen, MS, United States; dilution 1:250) overnight at 4°C and detected using a donkey anti-goat Alexa Fluor 488 (ab6881; abcam, Cambridge, United Kingdom) for 1 hour at room temperature. Followed by 1.5% normal horse blocking serum and 1.5% normal goat blocking serum in PBS for 30 minutes and incubated with primary antibody mouse anti-human CD34 (ab8536; abcam, Cambridge, United Kingdom; dilution 1:100) overnight at 4°C and detected using a goat anti-rabbit Alexa Fluor 594 (R37121; Invitrogen, MS, United States) for 1 hour at room temperature. And finally, using 1.5% normal horse blocking serum and 1.5% normal goat blocking serum in PBS for 30 minutes and incubated with primary antibody rabbit anti-human Von Willebrand (ab6994; abcam, Cambridge, United Kingdom; dilution 1:200) overnight at 4°C and detected using a goat anti-rabbit Alexa Fluor 647 (A21244; Invitrogen, MS, United States) for 1 hour at room temperature. Tissues were counter stained with DAPI and cover slipped.

For basophils and mast cells, tissues were stained for 2D7 (basophils), tryptase (mast cells) and CD125 (Supplementary Figure 2). CD125 staining was performed by using 3% normal horse blocking serum in PBS for 40 minutes and incubated with primary antibody goat anti-human CD125 (PA5-47340; Invitrogen, MS, United States; dilution 1:250) overnight at 4°C and detected using a donkey anti-goat Alexa Fluor 594 (A11058; Invitrogen, MS, United States) for 1 hour at room temperature. Followed by 1.5% normal horse blocking serum and 1.5% normal goat blocking serum in PBS for 30 minutes and incubated with primary antibody mouse anti-human 2D7 (ab155577; abcam, Cambridge, United Kingdom; dilution 1:250) overnight at 4°C and detected using a goat anti-mouse Alexa Fluor 488 (A11001; Invitrogen, MS, United States) for 1 hour at room temperature. And finally, using 1.5% normal horse blocking serum and 1.5% normal goat blocking serum in PBS for 30 minutes and incubated with primary antibody rabbit anti-human tryptase (ab134931; abcam, Cambridge, United Kingdom; dilution 1:250) overnight at 4°C and detected using a goat anti-rabbit Alexa Fluor 647 (A21244; Invitrogen, MS, United States) for 1 hour at room temperature. Tissues were counter stained with DAPI and cover slipped.

Tissues were imaged using the Light Microscope Basics Nikon Upright Eclipse Ni-U and analyzed using the NIS-Elements program. Cells of interest expressing any of the studied markers were counted manually and divided by the area of the region of interest and expressed as number per mm^2^). The precent changes from pre to post treatment were compared between the benralizumab and placebo groups using Mann Whitney U-test.

**Immunofluorescence Staining of PBMC for Flow Cytometric Analyses**

Peripheral blood mononuclear cells were stained with viability Dye (AF700) and incubated in Perm/Fix Buffer (BD Bioscience). Cells were then stained with antibodies for surface markers Lin-FTIC, CD45-APC-H7, CRTH2-BV421, CD127-BV605, and CD4-BV510, CD34-BV786 and CD125-APC or relevant isotype controls (BD Bioscience, Mississauga, ON, Canada; eBiosciences, San Diego, CA; R&D Systems, MN, USA). Lin-cocktail antibodies to (CD2, CD3, CD14, CD19, CD20 and CD94) plus FcεR1 excluded T and B lymphocytes, B-cells, monocytes, eosinophils, NK cells and basophils. After incubation, cells were washed and resuspended in fixed in PBS with 1% paraformaldehyde and analyzed by a FACS LSRII flow cytometer (BD Biosciences, CA, US) within 24 h. Gating in the lympho-mononuclear region (low side scatter/low forward scatter) and following acquisition of 300,000 events, data were analyzed using Flow-Jo software (Tree Star, CA, US) where CD4 T+ cells were defined as live, singlets CD45+Lin+CD4+ and CD4 T+ cells CD125+ (CD45+Lin+CD4+CD125+) (Supplementary Figure 4). Data are expressed as a proportion of the total CD45+ (white cell population) cells gated unless otherwise stated. The delta changes from pre to post treatment were compared between the benralizumab and placebo groups using Mann Whitney U-test.

***Supplementary Figure 1****. (A) Subgroup comparison of baseline EASI score in AD patients with low baseline blood eosinophils (≤0.3 cells/uL) compared to AD patients with high baseline blood eosinophils (>0.3 cells/uL). (B) Subgroup comparison of baseline blood eosinophil levels (cells/uL) in AD patients with asthma compared to AD patients without asthma. Data are shown as individual data with a mean and SEM. Groups are compared using a Mann-Whitnney U test. (C) Correlation graphs between baseline blood eosinophil levels (cells/uL) and baseline EASI in the population of AD patients with asthma. Data are shown as individual data and line of best fit. Correlation was calculated using Spearman correlation. ​*

******

***Supplementary Figure 2****. Shown are representative images of eosinophils measured in the papillary dermis by H&E stain, immunofluorescence staining of cells positive for EG2 (FITC), MBP (Cy5), CD125 (TRITC), cells double positive for EG2+CD125, cells double positive for MBP+CD125, triple positive for EG2+MBP+CD125 counter stained with DAPI, and immunofluorescence staining of eosinophil progenitors shown as CD34 (FITC)+ CD125 (TRITC)+ and Von Willebrand (Cy5) –ve and counter stained with DAPI. Skin samples were from lesion biopsies.*

**

***Supplmentary Figure 3****. Shown are repersentative images of basophils (2D7) and mast cells (tryptase) measured in the papillary dermis by immunofluorence staining of cells positive for 2D7 (FITC), tryptase (Cy5), cells double positive for 2D7 (FITC)+CD125 (TRITC), cells double positive for tryptase (Cy5)+CD125 (TRITC) and counter stained with DAPI. Skin samples were from lesion biopsies.*

******

***Supplementary Figure 4.*** *The effect of benralizumab compared to placebo on blood (A) CD4 T cells defined as CD45+ Lin+ CD4+; (B) CD4 T cells positive for IL-5Rα defined as CD125+CD45+ Lin+ CD4+. Data are shown as individual and mean (SEM), the delta change from pre to post treatment measurements were compared between benralizumab and placebo groups were analyzed by Mann-Whitey U test.*

******

***Supplementary Figure 5****. Flow cytometry gating of PBMC cells and the effect of benralizumab compared to blood on PBMC cells. All PBMC cells were gated as singlets, live cells, CD45+ cells. (A) CD4+ T cells were gated as CD45+ SSC^low^, Lin+, CD4+ and (B) CD4+ T cells CD125+ were gated as CD45+ SSC^low^, Lin+, CD4+ CD125+.*

**

***Supplementary Figure 6.*** *Clinical outcomes from the total patient population. (A) Eczema Area and Severity Index (EASI) (B) SCORing Atopic Dermatitis (SCORAD), (C) Investigator Global Assessment (IGA), (D) Dermatology Life Quality Index (DLQI) and (E) Patient Oriented Eczema Measure (POEM). Data are shown as group mean (SEM) and analyzed by Mann-Whitey U test.*

***Supplement Figure 7.*** *Clinical outcomes from patients with high baseline blood eosinophils (>0.3 cells/uL). (A) Eczema Area and Severity Index (EASI) (B) SCORing Atopic Dermatitis (SCORAD), (C) Investigator Global Assessment (IGA), (D) Dermatology Life Quality Index (DLQI) and (E) Patient Oriented Eczema Measure (POEM). Data are shown as group mean (SEM) and analyzed by Mann-Whitey U test.*
